# Effects of the Combination of Gliotoxin and Adriamycin on the Adriamycin-Resistant Non-Small-Cell Lung Cancer A549 Cell Line

**DOI:** 10.3390/md16040105

**Published:** 2018-03-27

**Authors:** Le Van Manh Hung, Yeon Woo Song, Somi Kim Cho

**Affiliations:** 1 School of Biomaterial Science and Technology, College of Applied Life Sciences, Jeju National University, Jeju 63243, Korea; manhhung.levan@gmail.com; 2 Faculty of Biotechnology, College of Applied Life Sciences, Jeju National University, Jeju 63243, Korea; syw1212@naver.com; 3 Subtropical/Tropical Organism Gene Bank, Jeju National University, Jeju 63243, Korea

**Keywords:** gliotoxin, NSCLC, adriamycin resistance, apoptosis

## Abstract

Acquired drug resistance constitutes an enormous hurdle in cancer treatment, and the search for effective compounds against resistant cancer is still advancing. Marine organisms are a promising natural resource for the discovery and development of anticancer agents. In this study, we examined whether gliotoxin (GTX), a secondary metabolite isolated from marine-derived *Aspergillus fumigatus*, inhibits the growth of adriamycin (ADR)-resistant non-small-cell lung cancer (NSCLC) cell lines A549/ADR. We investigated the effects of GTX on A549/ADR cell viability with the 3-(4,5-dimethylthiazol-2-yl)-2,5-diphenyltetrazolium bromide (MTT) assay and the induction of apoptosis in A549/ADR cells treated with GTX via fluorescence-activated cell sorting analysis, Hoechst staining, annexin V/propidium iodide staining, tetraethylbenzimidazolylcarbocyanine iodide (JC-1) staining, and western blotting. We found that GTX induced apoptosis in A549/ADR cells through the mitochondria-dependent pathway by disrupting mitochondrial membrane potential and activating p53, thereby increasing the expression levels of p21, p53 upregulated modulator of apoptosis (PUMA), Bax, cleaved poly (ADP-ribose) polymerase (PARP), and cleaved caspase-9. More importantly, we discovered that GTX works in conjunction with ADR to exert combinational effects on A549/ADR cells. In conclusion, our results suggest that GTX may have promising effects on ADR-resistant NSCLC cells by inducing mitochondria-dependent apoptosis and through the combined effects of sequential treatment with ADR.

## 1. Introduction

Non-small-cell lung cancer (NSCLC), which accounts for 85% of lung cancer cases, is one of the world’s leading causes of death, with an incidence of 1.8 million cases and 1.5 million deaths in 2012 [1,2]. Approximately 50% of patients diagnosed with NSCLC present with advanced disease (stage III or IV) that is not responsive to curative treatment. Patients with advanced NSCLC survive for only 9–12 months [3]. One explanation for these discouraging statistics is the development of drug resistance. Chemotherapy resistance has long constituted a major hurdle in the treatment of cancer, especially in NSCLC. This leads to increases in the drug dosage, which in turn increase the cytotoxicity and undesirable effects to normal cells/tissues. Chemotherapeutic resistance may occur as a consequence of several factors, including increased efflux capability of membrane P-glycoproteins such as multidrug resistance-related protein 1 (MRP1), multidrug resistance 1 (MDR1), or lung resistance protein (LRP); altered drug-metabolizing enzymes decreasing drug activation and increasing drug degradation; conjugation of the drug with increased glutathione; subcellular redistribution; drug interactions; elevated DNA repair; and failure of cells to undergo apoptosis [4]. Among these factors, apoptosis resistance is one of the most well studied, and it may be caused by the altered expression of genes involved in the apoptotic pathway [5]. Adriamycin (ADR), a topoisomerase II inhibitor belonging to the anthracycline family, is commonly used in cancer chemotherapy [6,7]. However, the use of ADR still has several limitations, such as acquired drug resistance, chronic cardiomyopathy, and congestive heart failure [8]. Moreover, the response rate of NSCLC to ADR is only 30–50% [9]. Thus, there is an urgent need to identify and develop an alternative approach to enhance the therapeutic outcome or increase the efficacy of this drug.

Developing anticancer drugs from natural sources is one of the most important strategies in the production of novel cancer therapies. Due to their extensive biodiversity, marine organisms represent a tremendous library of bioactive compounds. Marine-originated compounds have gained great interest as anticancer agents [10]. For example, Tuberatolide B isolated from the Korean marine algae *Sargassum macrocarpum* inhibits the proliferation of breast, lung, colon, prostate, and cervical cancer cells by disrupting the signal transducer and activator of transcription 3 (STAT3) signaling pathway [11]. Kempopeptin C isolated from a marine cyanobacterium from the Florida Keys inhibits the growth of invasive breast cancer by acting as a serine protease inhibitor [12]. Sinulariolide isolated from the soft coral *Sinularia flexibilis* decreases the epithelial–mesenchymal transition (EMT) in human bladder cancer cells [13]. Gliotoxin (GTX) is a secondary metabolite isolated from the marine-derived fungus *Aspergillus fumigatus*. It is an epipolythiodioxopiperazine characterized by a disulfide bridge across a piperazine ring (Please see Section 2.2) [14]. GTX has shown multiple biological effects, including antifungal [15], antiviral [16], anti-inflammatory [17], and antitumor activities in different cell lines [18,19]. In terms of its anticancer activity, GTX has shown great potential in targeting neurogenic locus notch homolog protein 2 (NOTCH2) [20,21], the Wnt/β-catenin pathway [18], farnesyltransferase, and geranylgeranyltransferase I [22]. However, the effects of GTX on chemoresistant cancer cells remain to be clarified.

In this study, we investigated the activities of GTX on the induction of apoptosis in ADR-resistant A549 cells using cell viability assays, detection of the formation of apoptotic bodies, cell cycle assay, mitochondrial membrane analysis, and western blotting. We also found that pretreatment with GTX for 12 h potentiated the effects of ADR, thereby reducing the effective concentration of ADR. Taken together, GTX shows promise as a potential therapeutic agent against chemoresistant NSCLC.

## 2. Results

### 2.1. Establishment of an ADR-Resistant Cell Line

**Figure 1:**
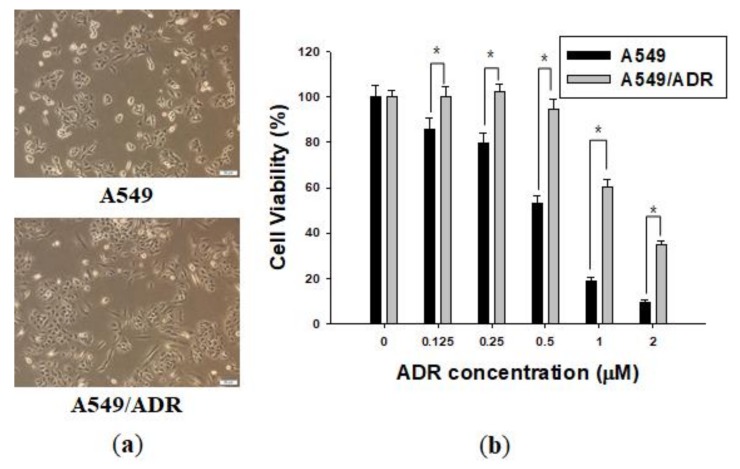
Characterization of the adriamycin (ADR)-resistant A549 cell line. (**a**) The morphologies of A549 and A549/ADR cells were observed under a microscope; (**b**) Effects of ADR on A549 and A549/ADR cells for 48 h. Cell viability was determined by the MTT assay. The results of independent experiments were averaged and are presented as percentage cell viability. Values represent means ± standard deviation (SD) (*n* = 3) (* *p* < 0.05).

Over 3 months, we established a cell line with acquired ADR resistance, generated from the A549 NSCLC cell line, named A549/ADR. The two cell lines were observed under a microscope and significant morphological differences were detected between the A549/ADR cell line and the original A549 line. The A549/ADR cells acquired an oval shape distinctly different from the epithelial-like shape of the parent cells (Figure 1a). The viability of A549 and A549/ADR cells was investigated using the 3-(4,5-dimethylthiazol-2-yl)-2,5-diphenyltetrazolium bromide (MTT) assay, which is based on the transformation of yellow tetrazolium salt MTT to purple formazan crystals by metabolically active cells. The IC_50_ values of ADR in A549 and A549/ADR cells were 0.55 and 1.40 μM at 48 h time point, respectively, for a resistance index of 2.55 for A549/ADR cells. Furthermore, 0.5 µM ADR did not decrease the proliferation of A549/ADR cells, whereas the viability of A549 cells gradually decreased after 48 h (Figure 1b).

### 2.2. GTX Overcame ADR Resistance in A549 NSCLC Cells

**Figure 2:**
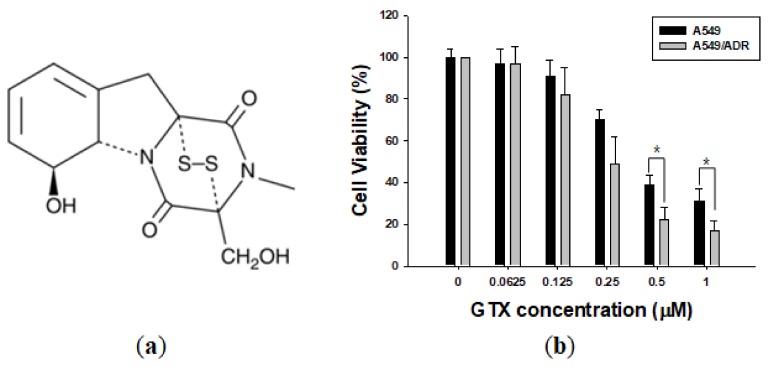
Gliotoxin (GTX) treatment reduces A549/ADR cell viability. (**a**) Chemical structure of GTX; (**b**) Effects of GTX on A549 and A549/ADR cells for 48 h. Cell viability was determined by the MTT assay. Results of independent experiments were averaged and are presented as percentage cell viability. Values represent means ± standard deviation (SD) (*n* = 3) (* *p* < 0.05).

Whereas ADR had strong cytotoxic effects on A549 cells but relatively weak effects on A549/ADR cells, GTX was effective in inhibiting the proliferation of both the parental A549 and resistant A549/ADR cells, with IC_50_ values of 0.40 and 0.24 µM, respectively. Interestingly, treatment of cells with GTX 0.5 and 1 μM significantly reduced the viability of A549/ADR cells than the viability of A549 cells. Apparently, there was no significant resistance against GTX compared to ADR. Moreover, GTX was more effective in inhibiting the proliferation of both cell lines than ADR (IC_50_ 0.40 and 0.24 vs. 0.55 and 1.40 µM) (Figure 2b).

### 2.3. GTX Induced Apoptosis in A549/ADR Cells

#### 2.3.1. GTX Induced Cell Cycle Arrest in A549/ADR Cells

**Figure 3:**
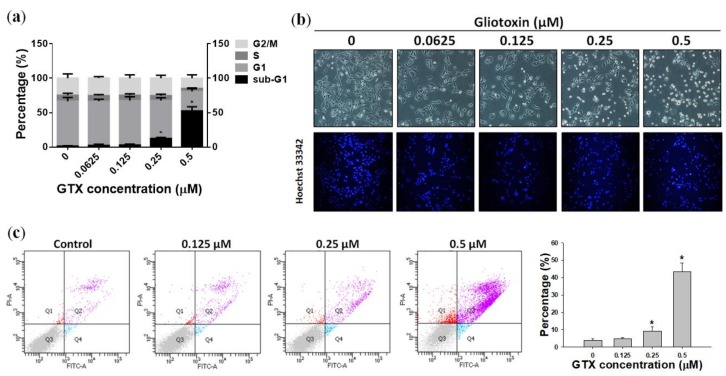
GTX treatment induces apoptosis in A549/ADR cells. (**a**) Cell cycle analysis of A549/ADR cells treated with GTX. Cells were seeded in 60-mm dishes and treated with different concentrations of GTX (0, 0.0625, 0.125, 0.25, and 0.5 µM) for 24 h. Cells were then stained with propidium iodide (PI) solution and analyzed by flow cytometry; (**b**) Cells were treated with increasing doses of GTX. After 24 h, apoptotic cells were detected by staining with Hoechst 33342 and observed under a fluorescence microscope; (**c**) Annexin V/PI staining analysis by flow cytometry. After cells were treated with 0, 0.125, 0.25, and 0.5 µM GTX for 24 h, they were stained with PI and annexin V-fluorescein isothiocyanate (FITC) together with binding buffer for 15 min before analysis. Values represent means ± standard deviation (SD) (*n* = 3) (* *p* < 0.05).

Propidium iodide (PI) staining and flow cytometry analysis were performed to investigate the cell cycle distribution of A549/ADR cells treated with 0.0625, 0.125, 0.25, and 0.5 µM GTX for 24 h (Figure 3a). Compared with the control sample, there was a dose-dependent increase of the sub-G1 population, from 1.37 to 52.49%, coupled with a decrease in the G1 population, from 65.41 to 28.44% (Figure 3a). This indicates that GTX-induced cell death of A549/ADR cells was mediated by sub-G1 cell cycle arrest and apoptosis.

#### 2.3.2. Hoechst 33342 Staining of A549/ADR Cells Treated with GTX

Chromatin condensation and apoptotic body formation, two characteristics of apoptosis, were investigated by Hoechst 33342 staining assay. Hoechst 33342 is a cell-permeable DNA stain that can be absorbed by both viable and dead cells. Viable cells with intact DNA show weak fluorescence signals, whereas cells undergoing apoptosis with condensed chromatin exhibit stronger fluorescence when observed under a fluorescence microscope. In this experiment, A549/ADR cells were treated with four concentrations of GTX for 24 h. As shown in Figure 3b, the number of A549/ADR cells with intense fluorescence signals increased in a dose-dependent manner, which indicates that apoptosis was the major cell death mechanism induced by GTX treatment.

#### 2.3.3. Annexin V/PI Staining

To continue to assess the lethality of GTX, A549/ADR cells were subjected to flow cytometry analysis after treatment with 0.125, 0.25, and 0.5 µM GTX for 24 h, and double stained with annexin V-fluorescein isothiocyanate (FITC) and PI solution. Detecting apoptosis with annexin V is based on the location of the membrane phospholipid phosphatidylserine (PS). In healthy cells, PS is located on the cytoplasmic side of the plasma membrane. However, in the early stages of apoptosis, PS translocates to the outer side of the membrane and can be detected by fluorescence-bound annexin V. The results are illustrated as a quadrant model with PI signal on the y-axis and annexin V intensity on the x-axis. The lower left quadrant shows the viable cells (negative for both PI and annexin V). The lower right quadrant shows the early apoptotic cells (PI negative, annexin V positive). The upper left quadrant shows necrotic cells (PI positive, annexin V negative), and the upper right quadrant shows the late apoptotic population (PI positive, annexin V positive). The results of quadrant statistical analysis showed that the number of annexin V-positive cells increased with increasing GTX dose. The apoptotic population in untreated cells was 3.73%, whereas it increased to 9.37% in 0.25 µM GTX-treated samples, and 43.37% in 0.5 µM GTX-treated samples (Figure 3c). Taken together with the previous experiments, these results indicate that GTX caused apoptosis in ADR-resistant A549 cells.

### 2.4. Disruption of Mitochondrial Membrane Potential by GTX in A549/ADR Cells

**Figure 4:**
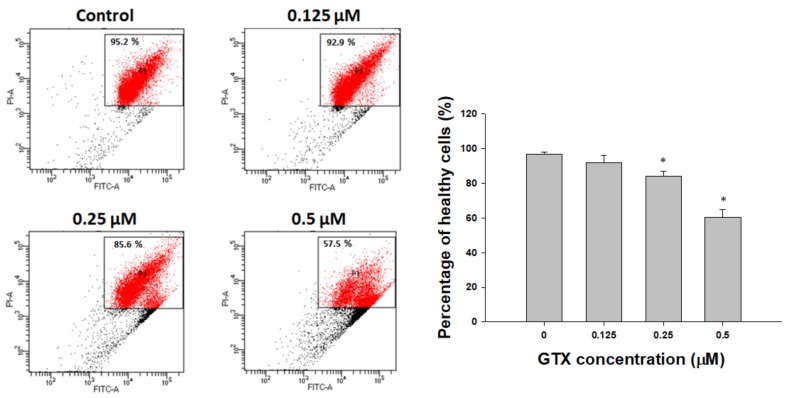
GTX attenuates mitochondrial membrane potential in A549/ADR cells. Cells were treated with three concentrations of GTX for 24 h, stained with tetraethylbenzimidazolylcarbocyanine iodide (JC-1)-FITC together with binding buffer, and analyzed by flow cytometry. Values represent means ± SD (*n* = 3) (* *p* < 0.05).

Mitochondrial membrane potential (ΔΨm) is an important indicator of cell condition, and depletion of ΔΨm is evidence of the early induction of apoptosis. Figure 4 shows that treatment with GTX (0–0.5 µM) increased the depolarization of mitochondrial membranes in A549/ADR cells, as indicated by the reduction in the level of ΔΨm (Figure 4). These data suggest that apoptosis caused by GTX treatment was induced through the intrinsic pathway.

### 2.5. Effects of GTX on the Expression of Apoptosis-Related Proteins

**Figure 5:**
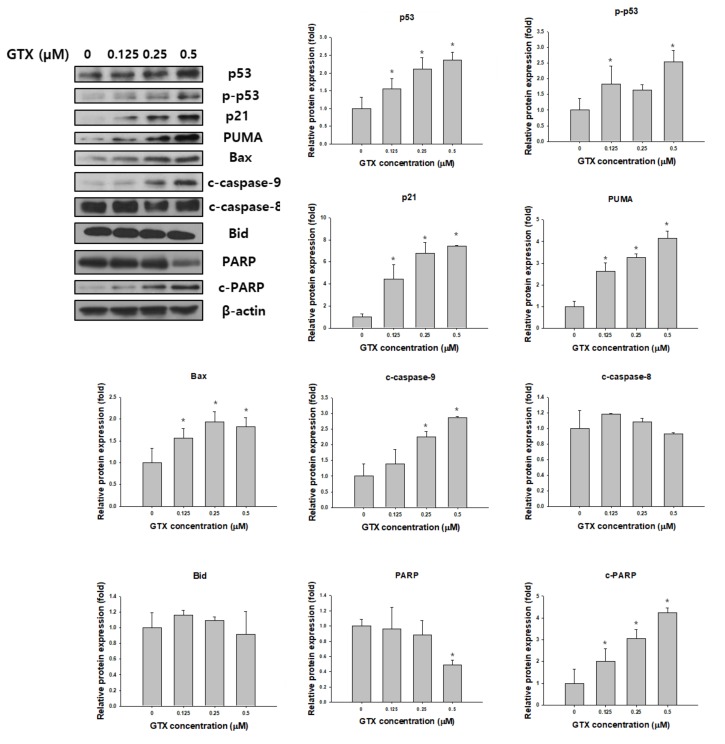
GTX induces A549/ADR cell death via the mitochondria-dependent pathway. Cells were treated with 0, 0.125, 0.25, and 0.5 µM GTX for 24 h and cell lysates were subjected to western blotting for various apoptosis-related markers including p53, phosphorylated p53 (p-p53), p21, PUMA, Bax, PARP, cleaved PARP (c-PARP), cleaved caspase-9 (c-caspase-9), cleaved caspase-8 and Bid. β-actin was used as a loading control. The graphs are the densitometric quantification of the western blotting result (* *p* < 0.05).

Western blot analysis was used to examine the expression of total p53, phosphorylated p53 (p-p53), p21, p53 upregulated modulator of apoptosis (PUMA), Bax, poly (ADP-ribose) polymerase (PARP), cleaved PARP, cleaved caspase-9, cleaved caspase-8 and Bid in A549/ADR cells after GTX treatment. β-actin was used as the loading control. As shown in Figure 5, GTX treatment significantly increased the expression of p53 and p-p53, and consequently, its downstream targets, including p21, PUMA, and Bax. Furthermore, the levels of cleaved PARP and cleaved caspase-9, two important apoptotic markers, were dose-dependently increased by GTX treatment (Figure 5).

### 2.6. GTX Potentiates the Effects of ADR on A549/ADR Cells

**Figure 6:**
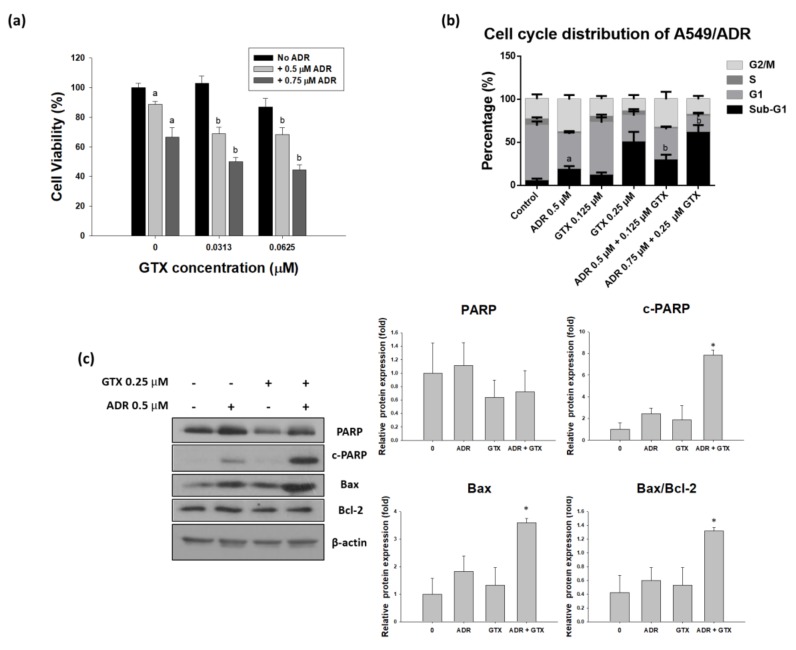
Pre-treatment with GTX for 24 h potentiates the effects of ADR in A549/ADR cells. A549/ADR cells were pretreated with GTX for 24 h and subsequently treated with ADR. The effects of the combination treatment were investigated by MTT assay (**a**); cell cycle analysis (**b**); and western blotting (**c**), the bar graphs represent the densitometric quantification of the western blotting result (* *p* < 0.05). Columns with different letters are significantly different between samples of the same ADR dose treatment (*p* < 0.05).

To determine the effects of the two drugs on ADR-resistant A549 cells, we treated these cells with GTX, ADR, or both (24 h GTX pretreatment) for 24 or 48 h. The MTT assay, cell cycle assay, and western blot analysis were performed to determine the ability of GTX to potentiate the effects of ADR in the ADR-resistant cell line. As shown in Figure 6a, the MTT assay indicated that A549/ADR cell viability was more severely decreased by the combinatorial treatment than with the single treatments. The CI values calculated by using Calcusyn (Biosoft, UK) were ranged between 0.8 and 1.3, which is comparable to the additive effect ranging from 0.9 to 1.1. Moreover, to study the cell cycle distribution after different treatments, we pretreated GTX for 24 h and then ADR for another 24 h, then we stained A549/ADR cells with PI solution and analyzed them with flow cytometry. The sub-G1 population was elevated from 11.44% for 0.125 µM and 50.02% for 0.25 µM GTX treatment alone to 29.09% and 61.16% for the combination treatment (Figure 6b). After we determined that GTX in combination with ADR potentiated the cytotoxicity in A549/ADR cells, we conducted western blot analysis to investigate changes in cleaved PARP and PARP, with β-actin as a loading control. We found that the level of PARP decreased in the GTX alone treatment and the expression of cleaved PARP in the GTX+ADR treatment group were higher than those in the individual treatments (Figure 6c). Moreover, the level of Bax was increased while the expression of Bcl-2 remained constant, this led to the increase of the Bax/Bcl-2 ratio, which plays an important role in apoptosis induction.

## 3. Discussion

Cancer raises many treatment challenges owing to the variable effectiveness of the primary therapies, surgery, radiation, and chemotherapy. Acquired drug resistance is considered a major barrier to a positive treatment outcome. ADR has been widely used to treat various kinds of solid tumors, including NSCLC. However, its efficacy is limited, as cancer cells often become resistant after a prolonged period of drug exposure. Natural resources have been used to develop antitumor agents [23]. In addition to terrestrial plants and microorganisms, marine species have emerged as a source of biologically active compounds for various purposes including anticancer effects. Among candidate marine-derived compounds, GTX is a fungal secondary metabolite isolated from the marine-derived *Aspergillus fumigatus*. Not much is known about the effects of marine products on drug resistant cancer cells. Previous studies have investigated the cytotoxicity of GTX in various cancer cell lines, however, the effects of GTX on chemoresistant cancer cells remains to be elucidated. In the present study, we examined the potential of GTX against drug resistant NSCLC A549/ADR cells and explored its underlying mechanism of action.

After testing a wide array of natural compounds in search of the most potent chemicals against both typical NSCLC A549 cells and ADR-resistant A549/ADR cells, we identified GTX as a compound that overcame ADR resistance and significantly inhibited both cell lines. Moreover, we reasoned that compounds with a lower IC_50_ are of interest for further study, as we can minimize the generic toxicity linked to ion transport. Although ADR had relatively low effectiveness in A549/ADR cells compared with A549 cells, GTX inhibited both cell lines in a similar manner. Interestingly, A549/ADR cell line was even more sensitive to GTX than A549 at high concentrations. This phenomenon could be explained by the fact that our A549/ADR has a higher expression level of multidrug resistance-associated protein 1 (MRP1) compared to the A549 cell line (data not shown). Previously, it has been reported that MRP1-overexpressing cells are more sensitive to a selective subset of chemical compounds due to collateral sensitivity [24,25]. In addition, De Groot et al. indicated that ADR-resistant small-cell lung cancer cells overexpressing MRP1 was more sensitive to indomethacin compared to the parental cell line [26]. In this study, the IC_50_ of GTX in A549 cells was 0.46 µM, which was more effective than in HeLa cervical cancer cells or SW1353 chondrosarcoma cells (both IC_50_ > 90 µM) [19], and equally potent in six breast cancer cell lines (IC_50_ ranging from 38 to 985 nM) [19]. Furthermore, GTX was also more effective than ADR (IC_50_ values were 0.40 and 0.24 compared with 0.55 and 1.40 µM, respectively). As the concentrations of IC_50_ or higher of ADR or GTX themselves exert dramatic effect on A549/ADR cells, we decided to choose the concentrations that are lower than the IC_50_ for our study on the combinational effect of ADR and GTX on A549/ADR cells.

Apoptosis is an ordered and well-arranged biological phenomenon that occurs under both physiological and pathological conditions. Apoptosis has been extensively studied for years in the cancer field. It is widely recognized that the induction of apoptosis is one of the most essential criteria in the development of antitumor agents [27]. In this study, we found that GTX inhibited the proliferation of A549/ADR cells by promoting mitochondria-mediated apoptosis. First, apoptosis induction by GTX was evidenced by the accumulation of populations of sub-G1 cells, the formation of apoptotic bodies, and increased numbers of annexin V-positive A549/ADR cells. Beside the apoptotic population, the percentage of necrotic cells were 0.3 ± 0.2, 0.5 ± 0.3, 0.9 ± 0.2, 4.5 ± 0.9 for GTX treatment at 0, 0.125, 0.25, 0.5 µM, respectively. In fact, apoptosis can be accompanied by necrosis, or necrosis can occur as the secondary phase after the apoptotic program has been completed [28]. To determine the type of apoptosis, we next used JC-1 staining to investigate whether the mitochondrial membrane potential was disrupted by GTX treatment. We observed a shift in fluorescence signal from red (~590 nm) to green (~529 nm), indicating the depolarization of the mitochondrial membrane as a result of mitochondria-dependent apoptosis. Additionally, we further explored the apoptotic pathway using western blotting. p53, which plays a crucial role in regulating the apoptotic pathway, was wild-type in both cell lines. As indicated by western blotting, p53 was upregulated, and the downstream targets of p53 were subsequently upregulated, including p21 (a cell cycle regulator), PUMA, and Bax (two pro-apoptotic proteins belonging to the Bcl-2 family). Moreover, the protein levels of proteolytically cleaved caspase-9 and cleaved PARP increased, clearly indicating the onset of apoptotic cell death. Furthermore, to exclude the possibility the extrinsic pathway is involved in GTX-induced apoptosis, we examined the expression level of cleaved caspase-8 and Bid. We observed that the expressions of both cleaved caspase-8 and Bid were not significantly altered by GTX treatment, therefore GTX induced apoptosis in A549/ADR via the intrinsic pathway.

A combination of natural compounds and approved chemotherapeutic agents to increase their efficacy has gained enormous attention in recent years. Several phytochemicals have been shown to elevate the outcome of ADR treatment, for example, schisandrin B reversed ADR resistance in MCF-7 breast cancer cells [29], neferine improved the effects of ADR on A549 lung adenocarcinoma cells [30], and resveratrol and didox increased the effects of ADR on HCT116 colorectal cancer cells [31]. Our findings suggest that GTX enhanced the cytotoxicity of ADR in an ADR-resistant A549 cell line. First, the MTT assay revealed that combined treatment resulted in a higher proportion of cell death. Then, by using a cell cycle distribution analysis, we demonstrated that sequential treatment with GTX and ADR elevated the sub-G1 population. Finally, western blot results showed that the combination of GTX and ADR enhanced the expression of significant protein in the apoptotic pathway, such as cleaved PARP and the Bax/Bcl-2 ratio. Moreover, the ability of GTX to reduce PARP protein level, which plays an important role in DNA damage repair pathway [32], could serve as a mechanism to potentiate the effect of DNA-damage-inducing agent ADR. 

## 4. Materials and Methods

### 4.1. Cell Line, Reagents, and Chemicals

F-12K, bovine serum albumin, trypsin/ethylenediaminetetraacetic acid, fetal bovine serum, and 100× antibiotic-antimycotic were purchased from Invitrogen (Carlsbad, CA, USA). Adriamycin (≥ 98% purity), gliotoxin (≥ 98% purity), hoechst 33342, dimethyl sulfoxide, MTT, PI, RNase A, and anti-β-actin antibodies were purchased from Sigma-Aldrich (St. Louis, MO, USA). Anti-cleaved PARP, -PARP, -Bax, -PUMA, -cleaved caspase-9, -p21, -p53, and -p-p53 (serine 15) antibodies were purchased from Cell Signaling Technology, Inc. (Beverly, MA, USA). Polyvinylidene fluoride membranes for western blotting were purchased from Millipore (Billerica, MA, USA). Mitochondrial membrane potential detection JC-1 kit and an annexin V-FITC apoptosis detection kit were purchased from BD Biosciences (San Jose, CA, USA).

### 4.2. Cell Culture

A549 human NSCLC and ADR-resistant NSCLC A549/ADR cells were cultured in F-12K medium supplemented with 10% heat-inactivated fetal bovine serum (FBS), 1% antibiotics at 37 °C in a CO_2_ incubator. All cells were gradually passaged when they reached 90% confluence. All cultures were maintained at 37 °C in a humidified incubator with 5% CO_2_. A549/ADR cells were obtained by using the dose-escalating method. Briefly, after examining the cell viability of A549 cells treated with different concentrations of ADR for 48 h, we chose 0.03 µM as our starting concentration for our procedure. A549 cells were maintained in medium containing ADR for two weeks with each concentration. ADR doses were gradually increased until it reached 0.5 µM, this process lasted for approximately three months. After that, we enriched three colonies that remained on the dish and performed MTT assay as well as western blotting for the MRP1 protein expression and chose the one with the highest cell viability and MRP1 expression as our ADR-resistant cell line.

### 4.3. Cell Viability Assay

The cell viability assay was performed as previously described [33]. Briefly, exponential-phase cells were seeded to 96-well plates (2.5 × 10^4^ cells/mL). After 24 h, cells were incubated in with various concentration of gliotoxin for 48 h. Then MTT agent was added into each well and incubated for 4 h at 37 °C. The MTT results were read immediately at 570 nm with a Sunrise microplate reader (Tecan, Salzburg, Austria). The percentage of viable cells was calculated based on the following formula: mean value of (control group–treated group)/control group × 100%. All results were assessed in triplicate at each concentration.

### 4.4. Flow Cytometry

PI and JC-1 staining for investigating cell cycle distribution and mitochondrial membrane potential were performed as previously described [33]. Briefly, A549/ADR cells were plated in 60 mm plates (15 × 10^4^ cells/plate) and treated with gliotoxin (0–0.5 µM) for 24 h. Then cells were harvested, washed with phosphate-buffered saline (PBS), and fixed in 70% ethanol. Before analysis, cells were treated with the solution containing 2 mM EDTA-PBS, RNase A (25 ng/mL) and propidium iodide (40 µg/mL). In terms of JC-1 staining assay, we harvested the GTX treated cells, then cells were washed with 1× assay buffer, stained with JC-1 for 15 min at 37 °C and washed again with 1× assay buffer. All analyses were performed using a FACSCalibur flow cytometer (BD Biosciences). Data were analyzed with CellQuest software (BD Biosciences). Each experiment was repeated at least three times.

### 4.5. Western Blot Analysis

Western blotting was performed as previously described [33]. Briefly, A549/ADR cells were plated in 60 mm dishes (15 × 10^4^ cells/plate). 24 h after being treated, cells were harvested and lysed in lysis buffer (20 nM Tris-HCl, 150 mM NaCl, 1 mM Na_2_EDTA, 1 mM EGTA, 1% NP-40, 2.5 mM sodium pyrophosphate, 1 mM β-glycerophosphate, 1 mM Na_3_VO_4_). Protein concentrations were measured and normalized using a BCA Protein Assay kit. Lysates were then separated by 10–15% sodium dodecyl sulfate-polyacrylamide gel electrophoresis and transferred onto a PVDF membrane using glycine transfer buffer. Membranes were then blocked for non-specific bindings by 5% skim milk solution. After that, membranes were incubated with primary antibodies overnight at 4 °C, followed by an additional 40 min incubation with secondary antibodies. The resultant membranes were analyzed using a BS ECL Plus kit (Biosesang Inc., Seongnam, Korea).

### 4.6. Statistical Analysis

Group comparisons were performed using Student’s *t*-test and one-way analysis of variance with Statistical Package for the Social Sciences software (SPSS v. 20.0, IBM Corp., Armonk, NY, USA). *p* < 0.05 was considered statistically significant.
